# Hydrogen Peroxide and Superoxide Anion Radical Photoproduction in PSII Preparations at Various Modifications of the Water-Oxidizing Complex

**DOI:** 10.3390/plants8090329

**Published:** 2019-09-05

**Authors:** Andrey Khorobrykh

**Affiliations:** Institute of Basic Biological Problems, FRC PSCBR RAS, Pushchino 142290, Moscow Region, Russia; andrewkhor@rambler.ru

**Keywords:** photosystem II, water-oxidizing complex, cytochrome b559 superoxide anion radical, hydrogen peroxide

## Abstract

The photoproduction of superoxide anion radical (O_2_^−•^) and hydrogen peroxide (H_2_O_2_) in photosystem II (PSII) preparations depending on the damage to the water-oxidizing complex (WOC) was investigated. The light-induced formation of O_2_^−•^ and H_2_O_2_ in the PSII preparations rose with the increased destruction of the WOC. The photoproduction of superoxide both in the PSII preparations holding intact WOC and the samples with damage to the WOC was approximately two times higher than H_2_O_2_. The rise of O_2_^−•^ and H_2_O_2_ photoproduction in the PSII preparations in the course of the disassembly of the WOC correlated with the increase in the fraction of the low-potential (LP) Cyt *b*_559_. The restoration of electron flow in the Mn-depleted PSII preparations by exogenous electron donors (diphenylcarbazide, Mn^2+^) suppressed the light-induced formation of O_2_^−•^ and H_2_O_2_. The decrease of O_2_^−•^ and H_2_O_2_ photoproduction upon the restoration of electron transport in the Mn-depleted PSII preparations could be due to the re-conversion of the LP Cyt *b*_559_ into higher potential forms. It is supposed that the conversion of the high potential Cyt *b*_559_ into its LP form upon damage to the WOC leads to the increase of photoproduction of O_2_^−•^ and H_2_O_2_ in PSII.

## 1. Introduction

Photosystem II (PSII) is a pigment–protein complex built into the thylakoid membrane. The main function of PSII is the light-induced oxidation of water to molecular oxygen with a transfer of electrons to the pool of plastoquinones. Recent crystallographic investigations of cyanobacterial PSII showed that a minimal structure capable of photosynthetic water oxidation and oxygen evolution (the so-called core complex of PSII) contains at least 20 protein subunits, 35 chlorophyll (Chl) molecules, 12 molecules of carotenoids, and at least 14–20 integral lipid molecules per monomer [1,2,3]. The light-induced charge separation with the formation of an oxidized primary electron donor, P_680_^+•^ (the strongest biological oxidant, with a redox potential of 1.1–1.27 V [4,5]), occurs in the photochemical reaction centre (RC) consisting of main proteins, D1 (PsbA) and D2 (PsbD), and cytochrome *b*_559_ (Cyt *b*_559_). P_680_^+•^ oxidizes TyrZ (tyrosine residue of D1 protein) with the formation of TyrZ^•^, which in turn takes an electron from the Mn_4_CaO_5_ cluster, the inorganic core of the water-oxidizing complex (WOC). The sequential absorption of photons and charge separation in the RC result in the formation of intermediate states (S_0_–S_4_) of the WOC, and the transition from S_4_ to S_0_ is accompanied by the oxygen release.

An integral part of the reaction centre is Cyt *b*_559_, which participates in redox reactions and, in comparison with other redox components of the RC, is not located inside the D1/D2 heterodimer. Cyt *b*_559_ can be found in at least four different redox forms: the Cyt *b*_559_ high-potential (HP) form (E = from + 350 mV to + 450 mV), in intermediate-potential (IP) form (E = from + 125 to +240 mV), a low-potential (LP) form (E = from −40 to + 80 mV) (see [6]), and in a so-called very low-potential (VLP) form with Em = from −150 to −200 mV [7,8]. The ratio of the redox forms of Cyt *b*_559_ PSII preparations depends on the structural integrity and composition of PSII. It was shown that the perturbation of the WOC led to the decrease of HP Cyt *b*_559_ and the increase of IP and LP Cyt *b*_559_ [9,10]. It was also shown that the conversion of HP Cyt *b*_559_ to the LP Cyt *b*_559_ could be induced by acidification of the medium [11]. Cyt *b*_559_ is assumed to participate in cyclic electron transfer, which is considered to be a protective mechanism against the photoinhibition of PSII, but this photoprotective role of Cyt *b*_559_ is debated [12,13,14]. It has been shown that Cyt *b*_559_ shows the following enzymatic properties: oxygen reductase, superoxide reductase, superoxide oxidase, and plastoquinol oxidase (see review in [15]).

When electrons from water pass into the electron transport chain of PSII, compounds with low redox potential are formed. They are considered to be the essential sources for the production of superoxide anion radicals (O_2_^−•^), which are subsequently converted to H_2_O_2_ and O_2_ via spontaneous or enzyme-catalyzed dismutation. Using a luminol–peroxidase method for the detection of H_2_O_2_, it was shown that the light-induced yield of the H_2_O_2_ in isolated oxygen-evolving PSII membrane fragments was slight (about 0.01 H_2_O_2_ molecules per RC and saturating flash) [16,17]. Possible donors of electrons to O_2_ can be the reduced forms of the primary electron acceptor pheophytin (Pheo^−^) [18], the primary (Q_A_^−^) and secondary (Q_B_^−^) quinone electron acceptors [19], plastosemiquinone (PQH^•^) (where O_2_^−•^ is produced via the proportion between plastoquinone (PQ) and plastoquinol (PQH_2_)) [20,21], and LP cytochrome Cyt *b*_559_ [22,23]. For a detailed description of O_2_^-•^ and H_2_O_2_ photoproduction in PSII, see also [24,25].

It was shown that the treatments leading to the perturbation of the PSII donor side increased H_2_O_2_ photoproduction [16,17,26,27]. It was assumed that the increase of H_2_O_2_ photoproduction in the PSII after a partial injury of the WOC could be associated with the replacement of the four-electron (with the release of O_2_) by the two-electron (with the production of H_2_O_2_) oxidation of water [16,27]. However, using isotope-labelled water in combination with a detection system for H_2_O_2_ showed that the oxygen in H_2_O_2_ formed during the illumination of NaCI-wash PSII membranes did not originate from water [26]. Thus, H_2_O_2_ photoproduction in PSII can occur both via the disproportionation of O_2_^−•^ formed as a result of the one-electron reduction of O_2_ on the PSII acceptor side and the incomplete photooxidation of water appearing after disturbance of the WOC.

In the present work, the effect of the step-by-step disassembly of the WOC on H_2_O_2_ and O_2_^−•^ photoproduction in PSII membrane fragments and core complexes was investigated. The light-induced formation of O_2_^−•^ and H_2_O_2_ in PSII was raised with the increasing destruction of the WOC. The comparison between H_2_O_2_ and O_2_^−•^ photoproduction in PSII preparations showed that O_2_^−•^ yield in all samples was approximately two times higher than H_2_O_2_. It is suggested that the stimulation of H_2_O_2_ photoproduction caused by the destruction of the WOC is mainly due to the acceptor side of PSII rather than the donor side via the enhancement of the O_2_^−•^ production, and Cyt *b*_559_ can play a crucial role in this.

## 2. Results

### 2.1. Functional Activity in PSII Preparations at Various Modifications of the WOC

**Figure 1:**
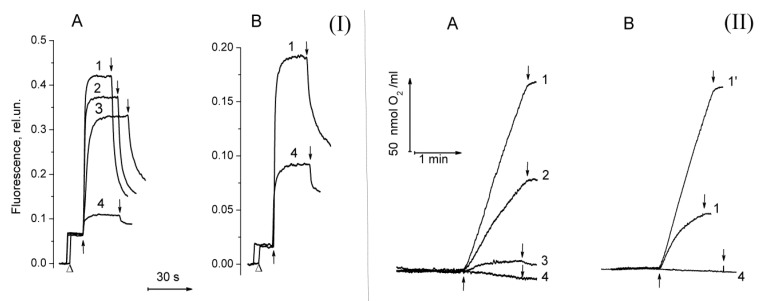
(**I**) Kinetics of photoinduced changes of chlorophyll fluorescence yield (ΔF) related to the photoreduction of the primary electron acceptor, Q_A_, in photosystem II (PSII) membranes fragments (**A**) and PSII core complexes (**B**) before (1) and after modification of the water-oxidizing complex caused by treatments with NaCl (2), CaCl_2_ (3), and NH_2_OH (4). The measurements of ΔF were done in a medium containing 50 mM MES–NaOH (pH 6.5), 35 mM NaCl and 0.4 M sucrose at a Chl concentration of 10 μg/mL. Δ, switching of the measuring light; ↑ and ↓, actinic light on and off, respectively. (**II**) Kinetics of oxygen evolution in PSII membranes (**A**) and PSII core complexes (**B**) before (1) and after modification of the water-oxidizing complex caused by treatments with NaCl (2), CaCl_2_ (3), and NH_2_OH (4). The measurements were made in the medium containing 50 mM MES–NaOH (pH 6.5), 35 mM NaCl, 0.4 M sucrose at a Chl concentration of 10 μg/ml for the PSII membranes and at 5 μg/ml for the PSII core complexes in the presence of 1 mM K_3_[Fe(CN)_6_] and 100 µM DCBQ. (1′)—oxygen evolution in the PSII core complexes was done in the presence of 5 mM CaCl_2_.↑ and ↓ – light (λ > 650 nm, 1500 μmol photon s^−1^ m^−2^) on and off, respectively.

The investigation of H_2_O_2_ and O_2_^−•^ photoproduction in PSII was carried out on the PSII membranes and the PSII core complexes with different degrees of damage to the WOC: untreated, and NaCl-, CaCl_2_-, and NH_2_OH-treated PSII. The step-by-step disassembly of the WOC led to the suppression of PSII activity (oxygen-evolving activity and photoinduced ΔF). The yield of photoinduced ΔF was decreased by 20% and 30% after NaCl and CaCl_2_ treatments of PSII membranes, respectively (Figure 1(I)A, curves 2 and 3). The complete removal of Mn ions from the WOC by NH_2_OH treatment led to a 5-fold decrease in the ΔF (Figure 1(I)A, curve 4) due to the loss of electron donation from the Mn-containing WOC to the PS II reaction centre (RC), which is in accordance with previous publications [28]. The photosynthetic oxygen evolution was more sensitive to the treatments in comparison with the ΔF (Figure 1(II) A). The rate of photosynthetic oxygen evolution in the untreated PSII membranes was about 600 µmol O_2_ (mg Chl h)^−1^. The treatment of PSII membranes with NaCl and CaCl_2_ resulted in a decrease in the rate of photosynthetic O_2_ evolution by 30% and 90%, respectively. The Mn removal from the WOC completely inhibited the oxygen-evolving activity of PSII and resulted in O_2_ photoconsumption which, as was shown earlier, was associated with both the photoformation of organic hydroperoxides on the donor side via a radical chain mechanism and with the photoproduction of H_2_O_2_ on the acceptor side of PSII [29,30,31].

The PSII core complexes showed maximal oxygen-evolving activity (about 1300 µmol O_2_ (mg Chl h)^−1^) only in the presence of exogenous Ca^2+^ (Figure 1(II)B, curves 1 and 1’). The CaCl_2_ dependence of the oxygen-evolving activity in the core complexes can be associated with the partial removal of PsbP and PsbQ proteins during ion exchange chromatography, since the concentration of MgSO_4_ used to elute the PSII cores was about 100 mM. It was shown that the release of PsbP and PsbQ proteins from the WOC suppressed PSII oxygen-evolving activity and the addition of CaCl_2_ reconstituted high rates of oxygen evolution in the PS II preparations deprived of these proteins [32]. Due to this reason, only NH_2_OH treatment was performed to modify the WOC in the PSII core complexes. In comparison with PSII membranes (where the release of Mn from the WOC resulted in a drastic decrease in the ΔF), the yield of ΔF in the Mn-depleted PSII core complexes was about two times less than in the untreated ones (Figure 1(I)B). A similar yield of ΔF was also observed in Mn-depleted PSII core complexes which were obtained by isolation from Mn-depleted PSII membranes. This may be due to the removal of a quinone from the Q_B_ site, since the Q_B_ quinone can release from its binding site during the isolation of PSII core complexes [33]. Even though the yield of ΔF in the Mn-depleted PSII core complexes was sufficiently high, the ability of the samples to perform photosynthetic oxygen evolution was completely lost (Figure 1(II)B, curve 4).

### 2.2. The Ratio in Redox Forms of Cyt b_559_ in PSII Preparations at Various Modifications of the WOC

In addition to the suppression of the PSII functional activity, the destruction of the WOC changed the ratio in redox forms of Cyt *b*_559_ in the PSII membranes (Table 1). The contents of HP, IP, and LP Cyt *b*_559_ in the untreated PSII membranes were 57%, 9%, and 34%, respectively. The treatment of PSII membranes with 1 M NaCl caused a slight decrease in the content of HP Cyt *b*_559_ and an increase of its IP form without changing the content of LP Cyt *b*_559_. A much stronger disturbance of the WOC induced by the treatment of PSII membranes with 1 M CaCl_2_ was accompanied by a significant decrease in the proportion of HP Cyt *b*_559_ and increase of IP and LP Cyt *b*_559_; thus, the ratio of the redox form of Cyt *b*_559_ in the samples was about 20% of the HP form, 35% of the IP form, and 45% of the LP form. In the Mn-depleted PSII membranes, most of Cyt *b*_559_ was in the LP (52%) and the IP (31%) forms, and only 17% was in the HP form. The similar interrelationship between the state of the WOC and the ratio in the redox forms of Cyt *b*_559_ in PSII preparations was shown previously [9,10]. In contrast to PSII membranes, untreated PSII core complexes contained about 12 % of HP Cyt *b*_559_, and this percentage did not change after the removal of Mn from the WOC. However, the untreated and Mn-depleted PSII core complexes considerably differed in the content of IP and LP Cyt *b*_559_: For the untreated samples, the contents of the IP and LP forms were 45% and 43%, respectively, while Mn-depleted samples contained 21% of the IP form and 67 % of the LP form (Table 1).

### 2.3. Photoproduction of H_2_O_2_ in PSII Preparations at Various Modifications of the WOC

**Figure 2:**
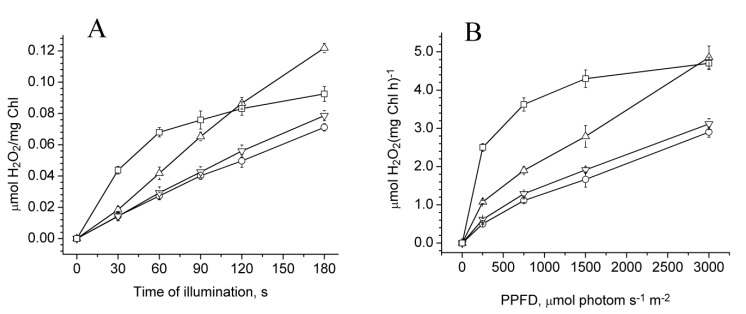
H_2_O_2_ photoproduction in PSII membranes before (◯) and after NaCl (▽), CaCl_2_ (△), and NH_2_OH (☐) treatments. (**A**) Dependence of H_2_O_2_ photoproduction in the PSII membranes on the duration of illumination (*λ* > 600 Hm, 1500 μmol photon s^−1^ m^−2^). (**B**) Dependence of H_2_O_2_ photoproduction in the PSII membranes on light intensity (the samples were illuminated at various light intensities for 1 min). The illumination of the samples was done in a medium containing 20 mM MES–NaOH (pH 6.5), 35 mM NaCl, and 0.4 M sucrose at 25 °C. The concentration of chlorophyll during illumination was 50 μg/mL.

Figure 2A illustrates the dependence of H_2_O_2_ photoproduction in the PSII membranes, varying in the degree of damage to the WOC, on the duration of illumination. The photoproduction of H_2_O_2_ by PSII membranes increased with the increasing destruction of the WOC. If, before treatments, the PSII membranes produced about 0.014 μmol H_2_O_2_ per mg Chl for 30 s of illumination (λ > 600 nm, 1500 µmol photon s^−1^ m^−2^), then after NaCl, CaCl_2_, and NH_2_OH treatments, the yield of H_2_O_2_ was 0.014, 0.018, and 0.045 μmol H_2_O_2_ per mg Chl, respectively. It appears from this that the Mn-depleted PSII membranes, in which the electron supply from water to the reaction centre was inhibited, produced three times more H_2_O_2_ than other samples. However, the capability of Mn-depleted PSII membranes to the light-induced production of H_2_O_2_ decreased during illumination. As a consequence, the amount of H_2_O_2_ produced by the Mn-depleted PSII membranes with 3 min of lighting was close to that generated by untreated samples. Ono and Inoue [34] showed that a gradual release of Mn from the WOC in the CaCl_2_-washed PSII membranes took place, and the Mn abundance in the samples decreased to about one half of the initial level after incubation in CaCl_2_-free medium at 0 °C under darkness for 7 h. In our case, the incubation time of the CaCl_2_-treated PSII membranes at 0 °C did not exceed 30 minutes, since a small aliquot of the samples was thawed for each series of measurements. In this regard, the number of reaction centres containing two manganese ions should be small based on the total number of reaction centres. Nevertheless, the CaCl_2_-treated samples containing about two Mn ions per RC were specially prepared. The rates of H_2_O_2_ and O_2_^−•^ photoproduction in these samples were two times higher than those of the CaCl_2_-treated PSII membranes containing four Mn ions per RC (data not presented).

**Figure 3:**
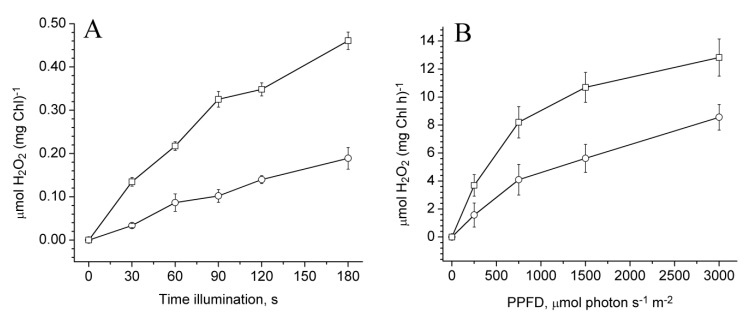
H_2_O_2_ photoproduction in untreated (◯) and Mn-depleted PSII core complexes (☐). (**A**) Dependence of H_2_O_2_ photoproduction in the PSII core complexes on the duration of illumination (*λ* > 600 Hm, 1500 μmol photon s^−1^ m^−2^). (**B**) Dependence of H_2_O_2_ photoproduction in the PSII core complexes on light intensity (the samples were illuminated at various light intensities for 1 min). The illumination of the samples was done in a medium containing 20 mM MES–NaOH (pH 6.5), 35 mM NaCl and 0.4 M sucrose at 25 °C. The concentration of chlorophyll during illumination was 50 μg/mL.

Figure 2B shows the dependence of the rate of H_2_O_2_ photoproduction by the PSII membranes on light intensity. The rate of H_2_O_2_ production was calculated by monitoring the concentration of H_2_O_2_ formed upon 1 min illumination of the samples. The rate of H_2_O_2_ photoproduction in untreated PSII membranes at 250 μmol photons m^−2^ s^−1^ was equal to 0.5 μmol H_2_O_2_ (mg Chl h)^−1^, and it increased two times after CaCl_2_ treatment of the PSII membranes and five times after Mn removal. The difference in the rate of H_2_O_2_ photoproduction between the untreated and Mn-depleted PSII membranes gradually decreased with increasing light intensity, to the extent that at the photosynthetic photon flux density (PPFD) of 3000 μmol of photons m^−2^ s^−1^, the rate of H_2_O_2_ photoproduction by Mn-depleted PSII membranes was only two times higher than in untreated ones (4.7 and 2.8 μmol H_2_O_2_ (mg Chl h)^−1^, respectively). At the same time, the difference in the rates of H_2_O_2_ production between untreated and NaCl- and CaCl_2_-treated PSII membranes upon the increase of PPFD was practically unchanged. Similar to PSII membranes, the removal of Mn clusters from the PSII core complexes stimulated the photoproduction of H_2_O_2_ (Figure 3). The rate of H_2_O_2_ photoproduction in Mn-depleted PSII core complexes calculated for 30 s after the start of continuous illumination (λ > 600 nm, 1500 µmol photon s^−1^ m^−2^) was four times higher than the untreated samples (16 μmol and 4 μmol H_2_O_2_ per mg Chl h, respectively). However, the suppression of H_2_O_2_ production in Mn-depleted PSII core complexes during illumination or at increasing light intensity occurred slower than in the Mn-depleted PSII membranes.

In addition to H_2_O_2_, other species of peroxides (such as organic hydroperoxides) also can be formed upon the illumination of PSII preparations, which is especially applicable to the Mn-depleted samples [29,30,31]. To obtain insight into the specificity of homovanilic acid (HVA) for other peroxide species, the reaction of the probe with two peroxides—m-chloroperbenzoic acid (MCPBA) as a model of a lipophilic hydroperoxide and tert-butyl hydroperoxide (TBHP) as a hydrophilic hydroperoxide—was examined. The addition of MCPBA or TBHP at a concentration even ten times higher than H_2_O_2_ resulted in only a slight increase in the fluorescence intensity of HVA, indicating that the contribution of hydroperoxides (which could be formed on the donor side of PSII) was negligible (Supplementary Materials Figure S1).

### 2.4. Photoproduction of O_2_^−•^ in PSII Preparations at Various Modification of the WOC

**Figure 4:**
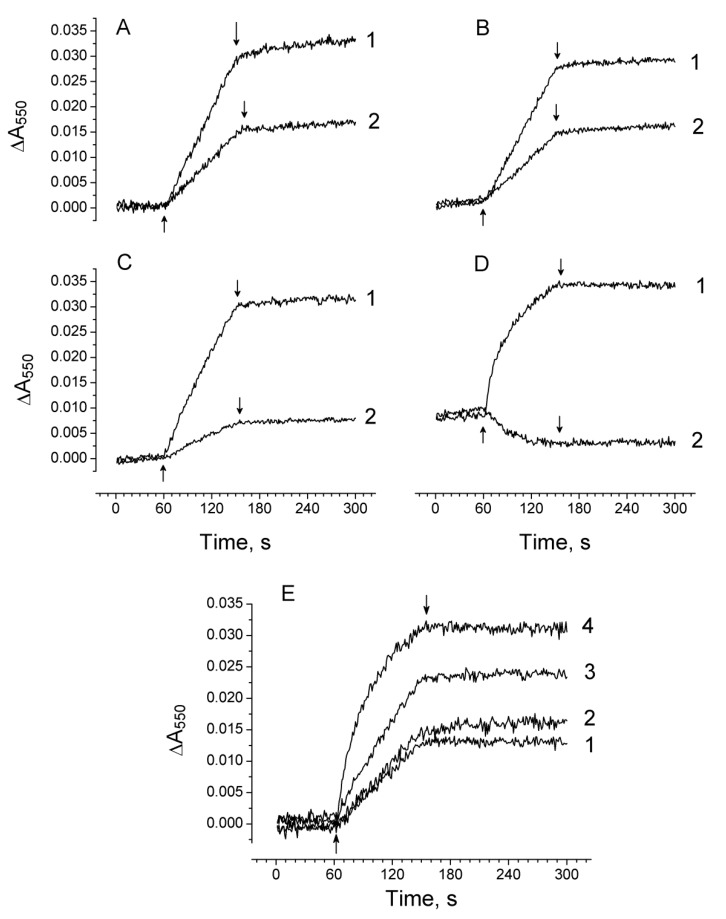
Kinetics of Cyt *c* photoreduction by PSII membranes before (**A**) and after modification of the water-oxidizing complex caused by treatments of NaCl (**B**), CaCl_2_ (**C**), and NH_2_OH (**D**). The measurements were done in the absence of additions (1) and after the addition of 50 Un/ml SOD (2). Reaction medium contained 50 mM MES–NaOH (pH 6.5), 35 mM NaCl, 0.4 M sucrose, and 10 μM Cyt *c*. The PSII membranes were illuminated (λ > 600 nm, 1500 µmol photon s^−1^ m^−2^) at chlorophyll concentration of 10 µg/ml. Up and down arrows indicate light on and off, respectively. (E) Kinetics of Cyt *c* reduction associated with the light-induced O_2_^−•^ formation in the PSII membranes before (1) and after modification of the water-oxidizing complex caused by treatments of NaCl (2), CaCl_2_ (3) and NH_2_OH (4). The kinetics was obtained by the subtraction of the kinetics of Cyt *c* photoreduction measured in the presence of SOD from that measured in the absence of SOD.

**Figure 5:**
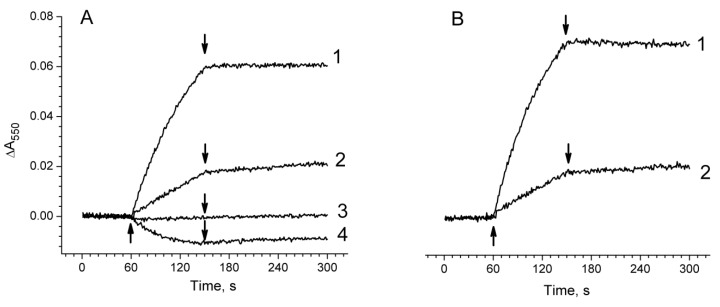
(**A**) Kinetics of Cyt *c* photoreduction by PSII core complexes before (1, 3) and after NH_2_OH treatment (2, 4). The measurements were done in the absence of additions (1, 2) and after the addition of 50 Un/ml SOD (3, 4). (**B**) Kinetics of Cyt *c* reduction associated with the light-induced O_2_^−•^ formation in the PSII core complexes before (1) and after Mn removal (2). The kinetics was obtained by the subtraction of kinetics of Cyt *c* photoreduction measured in the presence of superoxide dismutase (SOD) from that measured in the absence of SOD. Reaction medium contained 50 mM MES–NaOH (pH 6.5), 35 mM NaCl, 0.4 M sucrose and 10 μM Cyt *c*. The samples were illuminated (λ > 600 nm, 1500 µmol photon s^−1^ m^−2^) at chlorophyll concentration of 10 µg/ml. Up and down arrows indicate light on and off, respectively.

The main path of H_2_O_2_ production in PSII is the disproportion of superoxide anion radicals, which are from the one-electron reduction of O_2_ on the acceptor side of PSII. The photoproduction of O_2_^-•^ in the PSII preparations was investigated using Cyt *c*. To distinguish the photoreduction of Cyt *c* related to O_2_^−•^ from its reduction by reduced electron carriers on the acceptor side of PSII [35], the measurements were performed both in the absence and in the presence of superoxide dismutase (SOD). The photoreduction of Cyt *c* in untreated PSII membranes as well as in NaCl- and CaCl_2_-treated PSII membranes in the absence of SOD occurred with equal rates (Figure 4A–C, curve 1). The rate of Cyt *c* photoreduction in the Mn-depleted PSII membranes was much higher in comparison with other samples (especially during the first 10 seconds of illumination (Figure 4D, curve 1). The SOD added to the PSII membranes suppressed the Cyt *c* photoreduction and degree of the suppression depending on the destruction of the WOC (Figure 4A–D, curve 2). The inhibition of the Cyt *c* photoreduction with SOD was equal to 50%, 60%, and 76% in the untreated PSII, NaCl-, and CaCl_2_-treated PSII membranes, respectively. The addition of SOD completely suppressed Cyt *c* photoreduction by the Mn-depleted PSII membranes, and negative ΔA_550_ was observed (Figure 4D, curve 2) which, as was shown recently [35], is associated with photooxidation of reduced Cyt *c* on the donor side of PSII. Figure 4E shows the kinetics of the Cyt *c* photoreduction after the subtraction of the kinetics measured in the presence of SOD, which demonstrates O_2_^−•^-dependent Cyt *c* reduction. These data indicate that the increase in the damage to the WOC stimulates O_2_^−•^ photoproduction by PSII membranes. The removal of Mn clusters from the PSII core complexes also led to a significant increase in the rate of Cyt *c* photoreduction. However, in contrast to PSII membranes, the addition of SOD completely suppressed the Cyt *c* photoreduction both in untreated and Mn-depleted PSII core complexes (Figure 5A, curves 3 and 4), indicating that the samples were not capable of reducing Cyt *c* by electron carriers. Figure 5B shows the Cyt *c* reduction associated with the light-induced O_2_^−•^ formation in PSII core complexes. As can be seen from the figure, Mn removal from the WOC led to a significant (more than five times) stimulation of O_2_^−•^ photoproduction in PSII core complexes.

The addition of 20 µM diuron led to the almost complete suppression of H_2_O_2_ and O_2_^−•^ photoproduction in all the samples. This demonstrates that H_2_O_2_ and O_2_^−•^ photoproduction is linked to electron transport in PSII.

Table 2 shows the comparison in the rates of H_2_O_2_ and O_2_^−•^ photoproduction in PSII preparations at various modifications of the WOC. As illustrated above, the ability of the Mn-depleted PSII preparations to produce H_2_O_2_ and O_2_^−•^ was significantly decreased during illumination as a consequence of their sensitivity to light. Therefore, the rates were calculated for 30 s after the start of illumination (λ > 600 nm, 1500 µmol photon m^−2^ s^−1^) of the PSII preparations. The rate of light-induced formation of O_2_^−•^ and H_2_O_2_ in the PSII preparations rose with the increasing destruction of the WOC, and the photoproduction of O_2_^−•^ in all samples was almost two times higher than H_2_O_2_. The data suggest that all or most of the H_2_O_2_ comes from O_2_^−•^ dismutation, where two molecules of O_2_^−•^ form one peroxide molecule.

### 2.5. Effect of Exogenous Electron Donors on the Photoproduction of O_2_^−•^ and H_2_O_2_ in Mn-Depleted PSII Preparations

**Figure 6:**
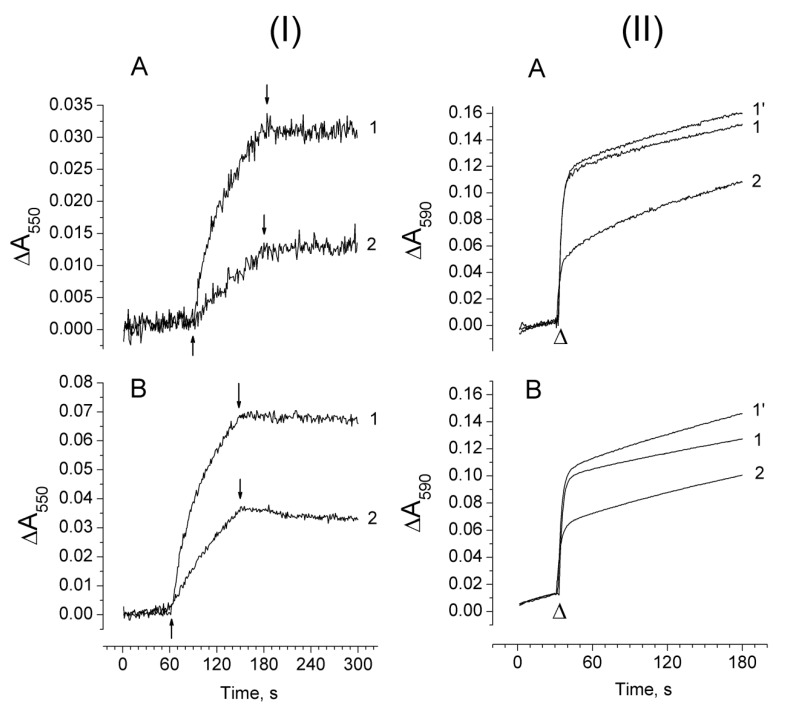
(**I**) Kinetics of Cyt *c* photoreduction related to the production of O_2_^−•^ in Mn-depleted PSII membranes (**A**) and Mn-depleted PSII core complexes (**B**) in the absence (1) and the presence of 50 µM DPC (2). ↑ and ↓ light on and off, respectively. (**II**) Absorption changes at 590 nm measuring H_2_O_2_ production in the Mn-depleted PSII membranes (**A**) and the Mn-depleted PSII core complexes (**B**) after illumination for 90 s (λ > 600 nm, 1500 µmol photon s^−1^ m^−2^) in the absence (1) and in the presence of 50 µM MnCl_2_ (2). 1′ −50 µM MnCl_2_ was added after the illumination of the samples. Chlorophyll concentration of the PSII membranes and core complexes was 50 µg Chl/ml and 20 µg Chl/ml, respectively. The light-induced yield of H_2_O_2_ in the samples was measured with 5 mM DMAB, 0.1 mM MBTH, and 3 unit/m1 horseradish peroxidase (HRP) (see Material and Methods). Δ—injection of HRP into the suspension of the samples.

Exogenous electron donors effectively restore photoinduced ΔF as a result of an increase in electron flow to the PSII reaction centre [28]. Figure 6I shows the Cyt c reduction associated with O_2_^−•^ photoproduction in Mn-depleted PSII membranes (Figure 6IA) and in Mn-depleted PSII core complexes (Figure 6IB) upon the addition of 50 µM diphenylcarbazide (DPC). The restoration of electron flow in the Mn-depleted PSII preparations by DPC resulted in a three-fold suppression of O_2_^−•^ photoproduction in PSII membranes, which was two-fold in PSII core complexes. The effect of the exogenous electron donor, Mn^2+^, on the photoproduction of H_2_O_2_ in the Mn-depleted PSII preparations was studied using an H_2_O_2_-dependent couple reaction between 3-methyl-2-benzothiazolinone hydrazone (MBTH) and 3-(dimethylamino) benzoic acid (DMAB) catalyzed by peroxidase. The use of another system for the determination of H_2_O_2_ was due to the fact that the electron donors used for the restoration of electron flow in the Mn-depleted PSII preparations affected the reaction of H_2_O_2_ with HVA. In addition to this, Mn^2+^ was used instead of DPC since DPC also affected the determination of H_2_O_2_ by this measuring system. MnCl_2_ (50 µM) added to the samples before illumination diminished the photoproduction of H_2_O_2_ in Mn-depleted PSII membranes and core complexes by 55% and 45%, respectively (Figure 6II, kinetics 1 and 2). Adding 50 µM MnCl_2_ to the samples after illumination had practically no effect on the light-induced yield of H_2_O_2_ (Figure 6II, kinetics 1′), indicating that MnCl_2_ did not affect the H_2_O_2_-dependent couple reaction between MBTH and DMAB as well, not leading to H_2_O_2_ decomposition.

## 3. Discussion

The obtained results demonstrate that the step-by-step disassembly of the WOC leading to the suppression of electron transport from the WOC to RC stimulates H_2_O_2_ and O_2_^−•^ photoproduction in PSII, and, among the samples, the Mn-depleted PSII preparations (which are not capable of water oxidation) show the highest rate of H_2_O_2_ and O_2_^−•^ photoproduction. The photoproduction of H_2_O_2_ in PSII can be associated with both the univalent reduction of O_2_ on the acceptor side to O_2_^−•^ (see [24,25]) and H_2_O_2_ formed on the donor side when the WOC is perturbed without the release of manganese [16,27]. In our case, the stimulation of H_2_O_2_ photoproduction in the PSII preparations induced by the injury of the WOC was mainly due to the increase in the O_2_^−•^ production on the acceptor side of PSII. This conclusion has been made based on the following observations: (1) The rate of O_2_^−•^ photoproduction was approximately two times higher than H_2_O_2_ (Table 2), and in the reaction dismutation, two O_2_^−•^ give the yield of one molecule of H_2_O_2_ (although the part of produced H_2_O_2_ can be oxidized by PSII during illumination, especially in the presence of exogenous Mn^2+^ [36,37]); (2) the PSII preparations deprived of Mn_4_CaO_5_ complex (when the water oxidation in PSII was lost entirely) showed maximal activity in O_2_^−•^ and H_2_O_2_ photoproduction. However, in comparison with the samples holding the Mn cluster, the capability of Mn-depleted PSII membranes for H_2_O_2_ photoproduction drastically decreased during illumination or at high light intensity. This behavior of Mn-depleted PSII membranes can be attributed to the deficiency of the electron source and high sensitivity of Mn-depleted PSII preparations to photoinhibition. The sources of electrons for P_680_^+•^ and TyrZ˙ in the absence of an Mn cluster can be chlorophylls and carotenoids (their photooxidation has been shown in several works [38,39,40,41,42]), lipids in the lipid belt around D1 and D2 (their presence in the RC has been demonstrated [2,43]), the amino acid residues involved in coordination of the Mn_4_CaO_5_ cluster [3], and His located in the vicinity of TyrZ. Apparently, the changes of the acceptor side caused by the modification of the WOC facilitate the photoproduction of O_2_^−•^. However, it cannot be excluded that the donor side of PSII also generates H_2_O_2_, especially in the case of CaCl_2_-treated PSII membranes [27,44], but its contribution seems negligible. In order to accurately estimate the contribution of the donor side, it is necessary to separate the H_2_O_2_ formed on the acceptor side from the donor side.

Pool PQ, pheophytin, Q_A_, and Cyt *b*_559_ are considered to be the primary sources involved in O_2_^−•^ and H_2_O_2_ photoproduction on the acceptor side (see [24,25]). It is worthwhile to consider the role of these cofactors in the enhancement of O_2_^−•^ and H_2_O_2_ photoproduction by PSII preparations after the destruction of the WOC.

The pool of PQ is shown to be involved in H_2_O_2_ formation within the thylakoid membrane [20,21]. The isolation of PSII preparations results in the deprivation of the PQ pool. It was shown that the PQ content was about 2.5 PQ/RC for PSII membranes [45], while the Q_B_ quinone could be release from its binding site during the isolation of PSII core complexes (these complexes did not emit the B-band arising from S_2_Q_B_ charge recombination, although the vacant Q_B_ pocket preserved a high affinity for 3-(3,4-dichlorophenyl)-1,1-dimethylurea (DCMU)) [33]. The analysis of PQ in the PSII core complexes isolated from cyanobacterium *Acaryochloris marina* MBIC 11017 showed that these complexes contained about 1.4 PQ per RC [46]. Since the increase of O_2_^−•^ and H_2_O_2_ photoproduction after damage to the WOC took place in both PSII membranes and core complexes, the participation of the PQ pool in O_2_^−•^ and H_2_O_2_ photoproduction seems to be vague, although we cannot exclude the possibility that some free PQ in PSII membranes could be involved O_2_^−•^ photoproduction. It was suggested [47] that O_2_^•−^ can be formed via the reduction of O_2_ by plastosemiquinones formed through the one-electron reduction of plastoquinone at the Q_B_ site and one-electron oxidation of plastoquinol by Cyt b_559_. Thus, it is possible that the involvement of PQ in O_2_^−•^ photoproduction induced by damage to the WOC occurs via its interaction with Cyt b_559_.

The redox potential of Pheo (its midpoint redox potential (Em) of the redox couple Pheo/Pheo^−^ at pH 7 is −610 mV [4,48]) favors the reduction of O_2_ to O_2_^−•^, since Em (O_2_/O_2_^−•^) is about −160 mV. According to Allakhverdiev and co-workers [49], the Em (Pheo/Pheo^−^) in PSII core complexes from *Synechocystis sp PCC 6803* was −525 mV for untreated and about −609 mV for Mn-depleted samples. Thus, the removal of manganese from the WOC shifts the Em (Pheo/Pheo^−^) towards negative values. It seems that this shift in the redox potential of Pheo would not lead to a significant increase of O_2_^−•^ and H_2_O_2_ photoproduction when the electron transport from the WOC to the RC was inhibited. In addition, the rate of H_2_O_2_ photoproduction in the Mn-depleted PSII preparations at low light intensity was five times higher than that in the samples containing “native” WOC, i.e., when the accumulation of the long-lived state of Pheo^−^ is less favorable. By contrast, the production of H_2_O_2_ in the Mn-depleted PSII preparations decreased with increasing light intensity or duration of illumination. It seems that the electron transfer directly from Pheo^−^ to O_2_ is not productive, although its reduction potential favors this reaction. Perhaps this is due to the recombination between P_680_^+^ and Pheo^−^ (which is less 5 ns) proceeding much faster than the electron transfer from Pheo^−^ to O_2_ or the difficulty of the formation of O_2_^−•^ within RC. If the enhancement of O_2_^−•^/H_2_O_2_ photoproduction in Mn-depleted PSII preparations is mainly associated with Pheo, then the restoration of electron flow in the samples by exogenous electron donors (DPC and Mn^2+^) would lead to the increase in production of O_2_^−•^ and H_2_O_2_. However, the restoration of electron flow in the samples diminishes the photoproduction of O_2_^−•^ and H_2_O_2_ (Figure 6). Nevertheless, the stimulation of O_2_^−•^/H_2_O_2_ photoproduction can be linked to the shift in the Em of Pheo/Pheo^−^ if it is assumed that the light-induced formation of O_2_^−•^ occurs in an aprotic environment, where Em for O_2_/O_2_^−•^ varies from −480 mV to −710 mV [50].

The Em for Q_A_/Q_A_^−^ in intact PSII preparations isolated from spinach has values of −84 mV [51], about −162 mV [52] or −146 mV [49]. The discrepancy in the values is attributed to the removal of bicarbonate from PSII during the measurement procedure of Em (Q_A_/Q_A_^−^) [53]. Thus, the redox potential of Q_A_ is not sufficient for the effective reduction of O_2_ to O_2_^−•^ (although it is assumed that this reaction occurs because the ratio between O_2_ and O_2_^−•^ is strongly shifted towards O_2_ [24]). In contrast to Pheo, the removal of the inorganic core of the WOC changes the Em (Q_A_/Q_A_^−^) by 150 mV to positive values [49,54,55]. Such a change of Em (Q_A_/Q_A_^−^) should lead to either to a slowdown or an interruption in the electronation of O_2_ by Q_A_^−^. However, the opposite tendency is observed: The destruction of the WOC accompanied by the shift of Em (Q_A_/Q_A_^−^) to positive values stimulates the light-induced formation of O_2_^−•^. This result may infer that the Q_A_ site is not directly responsible for the enhancement of O_2_^−•^, and H_2_O_2_ photoproduction observed after the damage to the WOC. DCMU prevents electron transport between Q_A_ and Q_B_ by the competitive binding of the herbicide molecule in the Q_B_ site on the reaction centre. The blocking of the electron transport between Q_A_ and Q_B_ facilitates the light-induced accumulation of Q_A_^−^, as evidenced by the acceleration of Fv rise upon the addition of diuron [56]. Thus, the 90% suppression of the photoproduction of O_2_^−•^ in the membranes and core complexes of PSII by diuron may also indicate that the main part of O_2_^−•^ is not formed on the Q_A_ site. It is probably true that this effect of diuron can be associated with the effects on the another components of the PSII reaction centre. It was shown that DCMU influenced the functioning of the WOC, the light-induced accumulation of reduced pheophytin [57], and the redox potential of HP Cyt *b*_559_ [58].

The involvement of Cyt *b*_559_ in O_2_ reduction is presented in several works (see [15]), and all of them confirm that only LP Cyt *b*_559_ can be involved in the reduction of O_2_ to O_2_^−•^. The redox potential of LP Cyt *b*_559_ varies from −40 mV to +80 mV (see [6]), which is not enough for the reduction of O_2_. However, it is assumed that Em (O_2_/O_2_^−•^) can be close to 0 mV if the concentration of O_2_ greatly exceeds the level of produced O_2_^−•^ [24]. Considering this fact, the LP Cyt *b*_559_ is capable of reducing O_2_ to O_2_^−•^. In addition to this, Cyt *b*_559_ can also exist in the VLP form, having more redox power for the reduction of O_2_ (the Em of the VLP form is from −150 to −200 mV [7,8]). The results presented here show that the increase in the fraction of LP Cyt *b*_559_ (including its VLP form) induced by damage to the WOC correlates with the rise of O_2_^−•^ and H_2_O_2_ photoproduction. It seems that the increase of the O_2_^−•^ photoproduction in PSII after the destruction of the WOC occurs due to the increase in the fraction of LP Cyt *b*_559_ and/or its VLP form. The suppression of O_2_^−•^ and H_2_O_2_ photoproduction in the Mn-depleted PSII preparations observed upon the addition of the exogenous electron donors (Figure 6) can be ascribed to the conversion of LP Cyt *b*_559_ to higher-potential forms. The conversion of LP Cyt *b*_559_ to IP and HP forms during the illumination of Mn-depleted PSII preparations in the presence of exogenous electron donors to PS II was shown previously by Mizusawa and co-workers [9].

Thus, the destruction of the WOC leading to the suppression of electron transport within the reaction centre of PSII promotes O_2_^−•^ and H_2_O_2_ photoproduction on the acceptor side of PSII through shifts in the redox potential of electron carriers of PSII. It seems that the conversion of HP and IP Cyt *b*_559_ to the LP form caused by the damage to the WOC makes a significant contribution to the enhancement of photoproduction of O_2_^−•^ and H_2_O_2_ in PSII. However, it is not improbable that a shift in the Em (Pheo/Pheo^−^) towards negative values may play a facilitating role in O_2_^−•^ photoproduction in terms of its formation in the aprotic environment. Perhaps the light-induced overproduction of O_2_^−•^/H_2_O_2_ associated with damage to the WOC may be a signal for the activation of processes necessary for the repair of damaged PSII, since the photoformation of O_2_^−•^ in native (undamaged) PSII is negligible.

## 4. Materials and Methods

### 4.1. Isolation of PS II Membranes and PSII Core Complexes

Oxygen-evolving PSII membrane preparations were isolated from spinach leaves according to the procedure in [59]. The samples were suspended in a medium containing 20 mM MES–NaOH (pH 6.5), 35 mM NaCl, 0.33 M sucrose, and 10% glycerol and stored at −76 °C. The isolation of PSII core complexes was performed according to the method in [60] with some modification: Bis-Tris buffer was replaced by MES. The concentration of chlorophyll (Chl) was measured as described previously [61]. The manganese content in PSII preparations was determined with an atomic absorption spectrophotometer equipped with a Kvant2A flame atomizer (Cortec, Russia).

### 4.2. Preparation of PSII Membranes with a Different Degree of Disassembly of the WOC and Mn-Depleted PSII Core Complexes

To obtain PSII membrane preparations with different degrees of disassembly of the WOC, the samples were treated by 1 M NaCl [62], 1 M CaCl_2_ [63], or 5 mM NH_2_OH [64]. According to the literature, the first treatment results in the depletion of two extrinsic proteins (PsbP and PsbQ) of the WOC (NaCl-treated PSII), while the incubation of the PSII preparations in the presence of 1 M CaCl_2_ releases all the external proteins (PsbP, PsbQ, and PsbO) from the WOC (CaCl_2_-treated PSII). Both these treatments do not extract manganese ions from the WOC, which suggests that the Mn cluster is relatively unaffected. The NH_2_OH treatment removes PsbP, PsbQ, and PsbO proteins and Mn ions from the WOC, but some amount of PsbO protein remains (Mn-depleted PSII).

Mn-depleted PSII core complexes were obtained by two approaches: (1) PSII core complexes were incubated in the presence of 5 mM NH_2_OH for 60 min, and then the samples were transferred to a Q-Sepharose column equilibrated with medium containing 20 mM MES-NaOH (pH 6.5), 35 mM NaCl, and 0.4 M sucrose with 0.03% (w/v) n-dodecyl-β-D-maltoside (medium A). After loading the samples, the column was washed with medium A with 1 mM ethylenediaminetetraacetic acid (EDTA) and then with medium A free from EDTA. The Mn-depleted PSII core complexes were eluted from the column by 100 mM MgSO_4_ being added into medium A; (2) Mn-depleted PSII core complexes were obtained from Mn-depleted PSII membranes in accordance with the procedure of isolation of PSII core complexes [60].

Atomic absorption spectroscopy measurements of the manganese content in PSII membranes showed that untreated and NaCl-treated PSII preparations had 4.2 ± 0.2 atoms of manganese per PSII reaction centre, while its content was 3.8 ± 0.1 and less than 0.1 Mn per RC in the CaCl_2_-treated and the Mn-depleted PSII membranes, respectively. The content of Mn ions in PSII core complexes was 3.9 ± 0.2 for untreated and close to 0 for the NH_2_OH-treated samples.

### 4.3. Measurements of Functional Activity of PSII Preparations

The functional activity of PSII preparations was estimated by photoinduced changes of chlorophyll fluorescence yield (ΔF) related to the photoreduction of the primary electron donor, Q_A_, and oxygen evolution measurements. The kinetics of photoinduced ΔF were measured in a 10 mm cuvette at room temperature by using an XE-PAM fluorometer (Walz, Germany). The photosynthetic oxygen evolution was measured in a temperature-controlled chamber by a Clark-type oxygen electrode (Hansatech Instruments, UK) at continuous illumination (λ > 600 nm, 1500 µmol photons s^−1^ m^−2^). The measurements were carried out at 25 °C in the presence of artificial electron acceptors for PSII 0.1 mM 2,6-dichloro-p-benzoquinone (DCBQ) and 1 mM K_3_[Fe(CN)_6_].

### 4.4. Determination of H_2_O_2_ Photoproduction by PSII Preparations

The photoproduction of H_2_O_2_ in PSII membranes or core complexes was studied using the fluorescent probe homovanilic acid (HVA). The method is based on the H_2_O_2_-dependent oxidation of HVA mediated by horseradish peroxidase (HRP) to a highly fluorescent dimer [65]. The PSII preparations, resuspended in medium containing 20 mM MES–NaOH (pH 6.5), 35 mM NaCl, and 0.4 M sucrose at 50 μg of Chl/ml, were illuminated or kept under darkness at 25 °C. Then, an aliquot (500 μl) of the samples was added into the same volume of the reaction medium containing 100 mM Hepes (pH 7.6), 600 μM HVA, and 2 Un/ml HRP. After 30 min incubation at 37 °C, the PSII membranes were centrifuged at 12,000 g for 2 min. The supernatant was collected, and its fluorescence spectrum (350−500 nm, λex = 312 nm) was recorded with a Cary Eclipse fluorescence spectrophotometer (Agilent, USA). To remove the PSII core complexes from the solution, they were loaded on an Amicon Ultra centrifugal filter (Ultracel 30K, Merck Millipore, Germany) and centrifuged at 5000 g for 15 min. The fraction passing through the filter (free from PSII core complexes) was collected, and the fluorescence spectrum was recorded. The difference between the fluorescence spectra of illuminated and unilluminated samples, designated as the “light minus dark” fluorescence spectrum, represented the light-induced formation of H_2_O_2_. The number of H_2_O_2_ formed under the illumination of the PSII preparations was calculated from the fluorescence intensity of HVA upon the addition of 5 µM H_2_O_2_. The effect of the exogenous electron donor, Mn^2+^, on the photoproduction of H_2_O_2_ in Mn-depleted PSII was examined by the method based on the oxidative coupling of 3-methyl-2-benzothiazolinone hydrazone (MBTH) and 3-(dimethylamino) benzoic acid (DMAB) in the presence of H_2_O_2_ peroxidase catalyzes, with the couple reaction between MBTH and DMAB with the formation of a deep purple compound having an absorption band between 575 and 600 nm with a peak at 590 nm [27,66]. The use of this method for detecting hydrogen peroxide was because Mn^2+^ did not interfere with the determination of H_2_O_2_ when using this system, while the presence of Mn^2+^ affected the detection of H_2_O_2_ by HVA. The measurements were performed as follows: 5 mM DMAB and 0.1 mM MBTH were added to the samples illuminated in the absence or the presence of 50µM MnCl_2_, then the change at 590 nm was recorded before and after the injection of HRP (3 Un/ml).

### 4.5. Determination of O_2_^−•^ Photoproduction by PSII Preparations

The light-induced generation of O_2_^−•^ in PSII was detected by cytochrome *c* (Cyt *c*) [67,68]. PSII membranes or core complexes were resuspended at 10 µg Chl/ml in a buffer solution containing 50 mM MES-NaOH(pH 6.5), 35 mM NaCl, 0.4 M sucrose, and 10 μM Cyt *c*. Kinetics of absorbance changes at 550 nm related to the reduction of Cyt *c* upon illumination of PSII preparations with red light (λ > 600 nm, 1500 µmol (photon) s^−1^ m^−2^) were measured in a 10 mm cuvette at room temperature using a spectrophotometer Agilent 8453 (USA). The rate of photoreduction of Cyt *c* was estimated by monitoring the concentration of reduced Cyt *c*. The amount of reduced Cyt *c* was calculated using the differential extinction coefficient between ferrocytochrome *c* and ferricytochrome *c* at 550 nm (21.1 mM^−1^).

### 4.6. Analysis of Redox Forms of Cyt b_559_ in PSII Preparations

Redox states of Cyt *b*_559_ in PSII preparations were determined by measuring the differential (reduced-minus-oxidized) absorption spectrum of Cyt *b*_559_ on a Shimadzu UV-1800 (Japan) spectrophotometer. To oxidize Cyt *b*_559_, 50 µM potassium ferricyanide was added. The reduction of the HP, IP, and LP (LP+VLP) forms of Cyt *b*_559_ was achieved by the stepwise addition of 5 mM hydroquinone, 5 mM sodium ascorbate, and sodium dithionite, respectively. After each addition of the redox agent, a differential absorption spectrum was recorded. The content of HP Cyt *b*_559_ was attributable to the spectra of Cyt *b*_559_ obtained upon the addition of hydroquinone to the samples with ferricyanide. The fraction of IP Cyt *b*_559_ was determined as the difference between the spectra of Cyt *b*_559_ reduced by ascorbate and the spectra of Cyt *b*_559_ reduced by hydroquinone, for the LP form of Cyt *b*_559_, and from the spectra of dithionite-reduced Cyt *b*_559_ were subtracted the ascorbate-reduced spectra of Cyt *b*_559_.

## Figures and Tables

**Table 1:** The ratio of Cyt *b*_559_ redox forms in PSII preparations after various modifications of the water-oxidizing complex.

Redox Forms of Cyt *b*_559_	PSII Membranes				PSII Core Complexes	
	Untreated	NaCl-Treated	CaCl_2_-Treated	Mn-Depleted	Untreated	Mn-Depleted
HP	56.6	43.7	19.8	17.4	12	11.8
IP	8.6	21.7	34.7	30.6	45.2	21
LP	34.8	34.6	45.5	52	42.8	67.2

**Table 2:** The rate of H_2_O_2_ and O_2_^−•^ photoproduction in PSII preparations after various modifications of the water-oxidizing complex. The rates were calculated for 30 s after the start of illumination (λ > 600 nm, 1500 µmol photon m^−2^ s^−1^) of the samples.

The Rate of Photoproduction, µmol (mg Chl h)^−1^	PSII Membranes				PSII Core Complexes	
	Untreated	NaCl-Treated	CaCl_2_-Treated	Mn-Depleted	Untreated	Mn-Depleted
H_2_O_2_	1.7 ± 0.36	1.7 ± 0.3	2.2 ± 0.25	5.25 ± 0.3	4.1 ± 0.6	16.2 ± 1.2
O_2_^−•^	2.7 ± 0.1	2.6 ± 0.2	5.3 ± 0.15	11.3 ± 0.3	6.9 ± 0.25	37.8 ± 0.5

## Supplementary Materials

The following are available online at https://www.mdpi.com/2223-7747/8/9/329/s1, Figure S1: Time course of homovanilic acid oxidation at 37 °C induced by 10 µM H_2_O_2_ (◯), 100 µM tert-Butyl hydroperoxide, and 100 µM m-Chloroperbenzoic acid (☐).

## References

[1] Loll B., Kern J., Saenger W., Zouni A., Biesiadka J. (2007). Lipids in photosystem II: Interactions with protein and cofactors. Biochim. Biophys. Acta.

[2] Guskov A., Kern J., Gabdulkhakov A., Broser M., Zouni A., Saenger W. (2009). Cyanobacterial photosytems II at 2.9-Å resolution and the role of quinones, lipids, channels and chloride. Nat. Struct. Mol. Biol..

[3] Umena Y., Kawakami K., Shen J.-R., Kamiya N. (2011). Crystal structure of oxygen-evolving photosystem II at a resolution of 1.9 Å. Nature.

[4] Klimov V.V., Allakhverdiev S.I., Demeter S., Krasnovsky A.A. (1979). Photoreduction of pheophytin in chloroplast photosystem II as a function of the redox potential of the medium. Dokl. Akad. Nauk SSSR.

[5] Ishikita H., Loll B., Biesiadka J., Saenger W., Knapp E.-W. (2005). Redox potentials of chlorophylls in the photosystem II reaction center. Biochemistry.

[6] Müh F., Zouni A., Cramer A., Kallas T. (2016). Cytochrome b559 in photosystem II. Cytochrome Complexes: Evolution, Structures, Energy Transduction, and Signaling.

[7] Shuvalov V.A., Schreiber U., Heber U. (1994). Spectral and thermodynamic properties of the two hemes of the D1D2cytochrome *b*-559 complex of spinach. FEBS Lett..

[8] Kaminskaya O., Kurreck J., Irrgang K.D., Renger G., Shuvalov V.A. (1999). Redox and spectral properties of cytochrome *b*559 in different preparations of Photosystem II. Biochemistry.

[9] Mizusawa N., Miyao M., Yamashita T. (1997). Restoration of the high-potential form of cytochrome b-559 by electron transport reactions through photosystem II in Tris-treated photosystem II membranes. Biochim. Biophys. Acta.

[10] Mamedov F., Gadjieva R., Styring S. (2007). Oxygen-induced changes in the redox state of the cytochrome b559 in photosystem II depend on the integrity of the Mn cluster. Physiol. Plant..

[11] Crofts J., Horton P. (1991). Dissipation of excitation energy by Photosystem II particles at low pH. Biochim. Biophys. Acta.

[12] Barber J., De Las Rivas J. (1993). A functional model for the role of cytochrome b559 in the protection against donor and acceptor side photoinhibition. Proc. Natl. Acad. Sci. USA.

[13] Thompson L.K., Brudvig G.W. (1988). Cytochrome b-559 may function to protect Photosystem II from photoinhibition. Biochemistry.

[14] Faller P., Fufezan C., Rutherford A.W., Wydrzynski T., Satoh K. (2005). Side path electron donors: Cytochrome b559, chlorophyll Z and β-carotene. Photosystem II: The Light-Driven Water: Plastoquinone Oxidoreductase.

[15] Pospíšil P. (2011). Enzymatic function of cytochrome b559 in photosystem II. J. Photochem. Photobiolb..

[16] Klimov V.V., Ananyev G.M., Zastryzhnaya O.M., Wydrzynski T., Renger G. (1993). Photoproduction of hydrogen peroxide in Photosystem II membrane fragments: A comparison of four signals. Photosynth. Res..

[17] Zastrizhnaya O.M., Khorobrykh A.A., Khristin M.S., Klimov V.V. (1997). Photoinduced production of hydrogen peroxide at the acceptor side of photosystem II. Biochemistry.

[18] Ananyev G.M., Renger G., Wacker U., Klimov V.V. (1994). The photoproduction of superoxide radicals and the superoxide dismutase activity of Photosystem II. The possible involvement of cytochrome *b*559. Photosynth. Res..

[19] Ananyev G., Wydrzynski T., Renger G., Klimov V. (1992). Transient peroxide formation by the manganese-containing redox-active donor side of photosystem II upon inhibition of O_2_ evolution with lauroylcholine chloride. Biochim. Biophys. Acta.

[20] Khorobrykh S.A., Ivanov B.N. (2002). Oxygen reduction in a plastoquinone pool of isolated pea thylakoids. Photosynth. Res..

[21] Khorobrykh S.A., Mubarakshina M., Ivanov B.N. (2004). Photosystem I is not solely responsible for oxygen reduction in isolated thylakoids. Biochim. Biophys. Acta.

[22] Kruk J., Strzałka K. (1999). Dark reoxidation of the plastoquinone-pool is mediated by the low potential form of cytochrome b559 in spinach thylakoids. Photosynth. Res..

[23] Pospišil P., Šnyrychova I., Kruk J., Strzałka K., Nauš J. (2006). Evidence that cytochrome *b*559 is involved in superoxide production in Photosystem II: Effect of synthetic short-chain plastoquinones in a cytochrome *b*559 tobacco mutant. Biochem. J..

[24] Pospísil P. (2009). Production of reactive oxygen species by photosystem II. Biochim. Biophys. Acta.

[25] Pospíšil P. (2012). Molecular mechanisms of production and scavenging of reactive oxygen species by photosystem II. Biochim. Biophys. Acta.

[26] Schröder W.P., Åkerlund H.E., Baltscheffsky M. (1990). Hydrogen Peroxide Production in Photosystem II Preparations. Current Research in Photosynthesis.

[27] Hillier W., Wydrzynski T. (1993). Increases in peroxide formation by the Photosystem II oxygen evolving reactions upon removal of the extrinsic 16, 22 and 33 kDa proteins are reversed by CaCl_2_ addition. Photosynth. Res..

[28] Klimov V.V., Allakhverdiev S.I., Shuvalov V.A., Krasnovsky A.A. (1982). Effect of extraction and re-addition of manganese on light reactions of photosystem II preparations. FEBS Lett..

[29] Khorobrykh S.A., Khorobrykh A.A., Klimov V.V., Ivanov B.N. (2002). Photoconsumption of oxygen in photosystem II preparations under impairment of the water-oxidizing complex. Biochemistry.

[30] Yanykin D.V., Khorobrykh A.A., Khorobrykh S.A., Klimov V.V. (2010). Photoconsumption of molecular oxygen on both donor and acceptor sides of photosystem II in Mn-depleted subchloroplast membrane fragments. Biochim. Biophys. Acta.

[31] Khorobrykh S.A., Khorobrykh A.A., Yanykin D.V., Ivanov B.N., Klimov V.V., Mano J. (2011). Photoproduction of catalase-insensitive peroxides on the donor side of manganese-depleted photosystem II: Evidence with a specific fluorescent probe. Biochemistry.

[32] Ghanotakis D.F., Babcock G.T., Yocum C.F. (1984). Calcium reconstitutes high rates of oxygen evolution in polypeptide depleted Photosystem II preparations. FEBS Lett..

[33] Sugiura M., Minagawa J., Inoue Y. (1999). Properties of chlamydomonas photosystem II core complex with a His-tag at the C-Terminus of the D2 protein. Plant. Cell Physiol..

[34] Ono T., Inoue Y. (1984). Ca^2+^ dependent restoration of evolving activity in CaCl_2_ washed PSII particles depleted of 33, 24 and 16 kDa proteins. FEBS Lett..

[35] Khorobrykh A.A., Yanykin D.V., Klimov V.V. (2018). Photooxidation and photoreduction of exogenous cytochrome c by photosystem II preparations after various modifications of the water-oxidizing complex. Photosynthetica.

[36] Boussac A., Picaud M., Etienne A.-L. (1986). Effect of potassium iridic chloride on the electron donation by Mn to photosystem II particles. Photobiochem. Photobiophys..

[37] Inoue H., Akahori H., Noguchi M. (1987). Activation of Electron Donation from Hydrogen Peroxide by Manganese in Non-oxygen evolving Photosystem II Particles. Plant. Cell Physiol..

[38] Klimov V.V., Shafiev M.A., Allakhverdiev S.I. (1990). Photoinactivation of the reactivation capacity of photosystem II in pea subchloroplast particles after a complete removal of manganese. Photosynth. Res..

[39] Telfer A., De Las Rivas J., Barber J. (1991). β-Carotene within the isolated photosystem II reaction centre: Photooxidation and irreversible bleaching of this chromophore by oxidised P680. Biochim. Biophys. Acta.

[40] Telfer A., Frolov D., Barber J., Robert B., Pascal A. (2003). Oxidation of the two *β*-carotene molecules in the photosystem II reaction center. Biochemistry.

[41] Hanley J., Deligiannakis Y., Pascal A., Faller P., Rutherford A.W. (1999). Carotenoid oxidation in photosystem II. Biochemistry.

[42] Tracewell C.A., Vrettos J.S., Bautista J.A., Frank H.A., Brudvig G.W. (2001). Carotenoid photooxidation in photosystem II. Arch. Biochem. Biophys..

[43] Mizusawa N., Wada H. (2012). The role of lipids in photosystem II. Biochim. Biophys. Acta.

[44] Yanykin D.V., Khorobrykh A.A., Terentyev V.V., Klimov V.V. (2017). Two pathways of photoproduction of organic hydroperoxides on the donor side of photosystem 2 in subchloroplast membrane fragments. Photosynth. Res..

[45] Kurreck J., Schödel R., Renger G. (2000). Investigation of the plastoquinone pool size and fluorescence quenching in thylakoid membranes and Photosystem II (PS II) membrane fragments. Photosynth Res..

[46] Yadav D.K., Prasad A., Kruk J., Pospíšil P. (2014). Evidence for the involvement of loosely bound plastosemiquinones in superoxide anion radical production in photosystem II. PLoS ONE.

[47] Tomo T., Okubo T., Akimoto S., Yokono M., Miyashita H., Tsuchiya T., Noguchi T., Mimuro M. (2007). Identification of the special pair of photosystem II in a chlorophyll d-dominated cyanobacterium. Proc. Natl. Acad. Sci. USA.

[48] Rappaport F., Guergova-Kuras M., Nixon P.J., Diner B.A., Lavergne J. (2002). Kinetics and pathways of charge recombination in photosystem II. Biochemistry.

[49] Allakhverdiev S.I., Tsuchiya T., Watabe K., Kojima A., Los D.A., Tomo T., Klimov V.V., Mimuro M. (2011). Redox potentials of primary electron acceptor quinone molecule (Qa) and conserved energetics of photosystem II in cyanobacteria with chlorophyll a and chlorophyll d. Proc. Natl. Acad. Sci. USA.

[50] Afanas’ev I.B. (1989). Superoxide Ion: Chemistry and Biological Implications.

[51] Krieger A., Rutherford A.W., Johnson G.N. (1995). On the determination of redox midpoint potential of the primary quinone electron acceptor, Q_A_, in photosystem II. Biochim. Biophys. Acta.

[52] Shibamoto T., Kato Y., Nagao R., Yamazaki T., Tomo T., Watanabe T. (2010). Species-dependence of the redox potential of the primary quinone electron acceptor Q_A_ in photosystem II verified by spectroelectrochemistry. FEBS Lett..

[53] Brinkert K., De Causmaecker S., Krieger-Liszkay A., Fantuzzi A., Rutherford A.W. (2016). Bicarbonate-induced redox tuning in Photosystem II for regulation and protection. Proc. Natl. Acad. Sci. USA.

[54] Krieger A., Weis E. (1992). Energy-dependent quenching of chlorophyll-a- fluorescence: The involvement of proton-calcium exchange at photosystem II. Photosynthetica.

[55] Johnson G.N., Rutherford A.W., Krieger A. (1995). A change in the midpoint potential of the quinone Q_A_ in Photosystem II associated with photoactivation of oxygen evolution. Biochim. Biophys. Acta.

[56] Hsu B.D., Lee J.Y., Pan R.L. (1986). The two binding sites for DCMU in photosystem II. Biochem. Biophys. Res. Commun..

[57] Klimov V.V., Shuvalov V.A., Heber U. (1985). Photoreduction of pheophytin as a result of electron donation from the water-splitting system to Photosystem-II reaction centers. Biochim. Biophys. Acta.

[58] Kaminskaya O., Shuvalov V.A., Renger G. (2007). Evidence for a novel quinone-binding site in the Photosystem II (PS II) complex that regulates the redox potential of cytochrome b559. Biochemistry.

[59] Ford R.C., Evans M.C.W. (1983). Isolation of a photosystem 2 preparation from higher plants with highly enriched oxygen evolution activity. FEBS Lett..

[60] Van Leeuwen P.J., Nieveen M.C., van de Meent E.J., Dekker J.P., van Gorkom H.J. (1991). Rapid and simple isolation of pure photosystem II core and reaction center particles from spinach. Photosynth. Res..

[61] Lichtenthaler H.K. (1987). Chlorophylls and carotenoids: Pigments of photosynthetic biomembranes. Methods Enzymol..

[62] Miyao M., Murata N. (1983). Partial disintegration and reconstitution of the photosynthetic oxygen evolution system. Binding of 24 kilodalton and 18 kilodalton polypeptides. Biochim. Biophys. Acta.

[63] Ono T., Inoue Y. (1983). Mn-preserving extraction of 33-, 24- and 16 kDa proteins from O_2_-evolving PS II particles by divalent salt-washing. FEBS Lett..

[64] Tamura N., Cheniae G.M. (1987). Photoactivation of the water-oxidizing complex in Photosystem II membranes depleted of Mn and extrinsic proteins. I. Biochemical and kinetic characterization. Biochim. Biophys. Acta.

[65] Ruch W., Cooper P.H., Baggiolini M. (1983). Assay of H_2_O_2_ production by macrophages and neutrophils with homovanillic acid and horse-radish peroxidase. J. Immunol. Methods.

[66] Ngo T.T., Lenhoff H.M. (1980). A sensitive and versatile chromogenic assay for peroxidase and peroxidase-coupled reactions. Anal. Biochem..

[67] Fridovich I. (1970). Quantitative aspects of the production of superoxide anion radical by milk xanthine oxidase. J. Biol. Chem..

[68] Chen G.X., Kazimir J., Cheniae G.M. (1992). Photoinhibition of hydroxylamine extracted photosystem II membranes: Studies of the mechanism. Biochemistry.

