# Correction: Khorobrykh, A. Hydrogen Peroxide and Superoxide Anion Radical Photoproduction in PSII Preparations at Various Modifications of the Water-Oxidizing Complex. *Plants* 2019, *8*, 329

**DOI:** 10.3390/plants10020187

**Published:** 2021-01-20

**Authors:** Andrey Khorobrykh

**Affiliations:** Institute of Basic Biological Problems, FRC PSCBR RAS, Pushchino 142290, Moscow Region, Russia; andrewkhor@rambler.ru

In the original article, there was a mistake in the legend for ** Figure 5 ** [1]. There is an inaccuracy concerning of the numbering of the curves. The correct legend appears below.

(**A**) Kinetics of Cyt *c* photoreduction by PSII core complexes before (2, 3) and after NH_2_OH treatment (1, 4). The measurements were done in the absence of additions (1, 2) and after the addition of 50 Un/ml SOD (3, 4). (**B**) Kinetics of Cyt *c* reduction associated with the light-induced O_2_^−•^ formation in the PSII core complexes before (2) and after Mn removal (1). The kinetics was obtained by the subtraction of kinetics of Cyt *c* photoreduction measured in the presence of superoxide dismutase (SOD) from that measured in the absence of SOD. Reaction medium contained 50 mM MES–NaOH (pH 6.5), 35 mM NaCl, 0.4 M sucrose and 10 µM Cyt *c*. The samples were illuminated (λ > 600 nm, 1500 µmol photon s^−1^ m^−2^) at chlorophyll concentration of 10 µg/mL. Up and down arrows indicate light on and off, respectively.

The authors apologize for any inconvenience caused and state that the scientific conclusions are unaffected. The original article has been updated.
